# Sustained-Release Vildagliptin 100 mg in Type 2 Diabetes Mellitus: A Review

**DOI:** 10.7759/cureus.39204

**Published:** 2023-05-18

**Authors:** GR Sridhar, Kaushik Pandit, Sona Warrier, Ashish Birla

**Affiliations:** 1 Endocrinology, Endocrine and Diabetes Centre, Visakhapatnam, IND; 2 Endocrinology, Diabetes and Metabolism, Belle Vue Clinic, Kolkata, IND; 3 Endocrinology, Diabetes and Metabolism, Institute of Post Graduate Medical Education & Research, Kolkata, IND; 4 Scientific Services, USV Pvt. Ltd., Mumbai, IND

**Keywords:** t2dm, type 2 diabetes mellitus, adherence, sustained release, vildagliptin, dpp4 inhibitors, diabetes

## Abstract

Dipeptidyl peptidase-4 inhibitors (DPP4Is) were introduced into the management of type 2 diabetes mellitus (T2DM) as they are insulinotropic and have no inherent risk of hypoglycemia and no effect on body weight. Currently, 11 drugs in this class are available for the management of diabetes. Although they have a similar mechanism of action, they differ from one other in their binding mechanisms, which influences their therapeutic and pharmacological profiles. Vildagliptin's overall safety and tolerability profile was comparable to placebo throughout clinical studies, and real-world data in a large group of T2DM patients corroborated this finding. Therefore, DPP4Is like vildagliptin is a secure alternative for treating patients with T2DM. Vildagliptin treatment given as a once-daily (QD) 100 mg sustained release (SR) formulation fits the criteria of adherence and compliance. This SR formulation, given once daily has the potential to provide glycemic control like the vildagliptin 50 mg twice-daily (BD) formulation. This comprehensive review discusses the journey of vildagliptin as 50 mg BD therapy as well as 100 mg SR QD therapy.

## Introduction and background

The enzyme dipeptidyl peptidase-4 (DPP4) plays a key role in regulating glucagon-like peptide 1 (GLP-1), an incretin hormone [1]. The GLP-1, a peptide secreted proximally in the small intestine, enhances glucose-induced insulin secretion and suppresses glucagon secretion. Since it can regulate fasting plasma glucose in type 2 diabetes mellitus (T2DM), it was considered to have a role in the treatment of T2DM. The DPP4 enzymes are shown to cleave GLP-1 from the amino terminus of the intact peptide. Since GLP-1 has a short half-life physiologically, inhibitors of DPP4 were developed to reduce the degradation of GLP-1, which would be therapeutically beneficial. Therefore, DPP4 inhibitors (DPP4Is) were introduced into the management of T2DM [1].

Dipeptidyl peptidase-4 inhibitors are insulinotropic, have no inherent risk of hypoglycemia, and have no effect on body weight [2]. They are known to enhance physiological GLP-1 levels [3], which delay stomach emptying time and reduce appetite. This property makes them weight-neutral. Dipeptidyl peptidase-4 inhibitors also boost insulin production while decreasing glucagon secretion in response to increased plasma glucose. These results were validated by clinical studies in T2DM, where DPP4Is exhibited significant reductions in HbA1c in addition to other pleiotropic benefits. In response to hyperglycemia, DPP4Is boost insulin production while decreasing glucagon secretion. The benefits of DPP4Is include a good adverse-effect profile, a complementary mechanism of action with other anti-diabetic drugs, and a neutral effect on weight, offering a beneficial choice in the drug therapy of diabetes.

Currently, 11 drugs in this class are available for the management of diabetes. Although they have a similar mechanism of action, they differ in their binding mechanisms, which influences their therapeutic and pharmacological profiles. Dipeptidyl peptidase-4 inhibitors are increasingly prescribed because of their efficacy and lack of undesirable effects compared to the earlier group of available agents [4].

## Review

The indications for gliptins are given in Table 1.

**Table 1:** Indications for gliptins HbA1c: Glycated hemoglobin, SU: Sulfonylurea, T2DM: Type 2 diabetes mellitus, TZD: Thiazolidinedione

Line of treatment	Indications
First	Patients with T2DM with HbA1c 7%
Second	For uncontrolled T2DM with HbA1c>7%, one of the following medications—metformin, SU, TZD, alfa-glucosidase inhibitors, or meglitinide—should be used as an add-on therapy in T2DM patients.
Third	In T2DM patients receiving combination therapy, two of the following drugs are included: metformin, SU, TZD, an alpha-glucosidase inhibitor, meglitinide

Vildagliptin in the management of T2DM

India’s central drug standard control organization (CDSCO) approved vildagliptin in 2008 [5]. A single dose of 50 mg vildagliptin provides ≥80% DPP4 inhibition for 12 hours; a bolus of 100 mg vildagliptin confers the same DPP4 inhibition for 15 to 16 hours [6]. When combined with metformin, pioglitazone, or insulin in T2DM, vildagliptin lowered glycated hemoglobin (HbA1c) levels [5].

Molecular interaction of vildagliptin with DPP4 enzyme

Vildagliptin extends the duration of meal-induced elevations in GLP-1 and gastric inhibitory polypeptide (GIP) by inhibiting the DPP4 enzyme. The physiological meal-induced elevation of GLP-1 and GIP levels is generally prolonged by all DPP4Is. However, the duration of this effect depends on the degree of DPP4 inhibition by each agent. Fifty percent inhibition would extend the half-lives of GLP-1 and GIP 2-fold; 90% inhibition would extend them 10-fold; and 95% inhibition, 20-fold. With 100% inhibition of DPP4, the half-lives of GLP-1 and GIP would be determined only by their renal clearance [7].

Vildagliptin is a slow substrate for DPP4 that inhibits GLP-1 and GIP inactivation. At clinically recommended doses, vildagliptin maintains pancreatic levels of GLP-1 and GIP levels above the threshold activity for 24 hours. Vildagliptin is a chemical compound with a nitrile group that rapidly establishes a covalent bond with the DPP4 catalytic site to produce an imidate group. The hydrolysis of the imidate group is facilitated by this bond, which also stabilizes vildagliptin in the DPP4 catalytic site. With a half-life (T1/2) of around an hour, inactive vildagliptin then gradually separates from the catalytic site. Vildagliptin forms a covalent bond with the DPP4 catalytic site, blocking the enzyme's ability to interact with other substrates. Another vildagliptin molecule binds with the catalytic site right away following dissociation. A plasma level of 50 nM is sufficient to maintain this block, which leads to a complete blockage of DPP4 activity for the duration that vildagliptin levels can associate with the catalytic site (and so block it) [8].

Efficacy and safety of vildagliptin

Vildagliptin is effective, has a low risk of hypoglycemia, is weight neutral, and has no increased risk of adverse cardiovascular effects [9]. It has been shown to be effective in both newly diagnosed diabetes [10] and those with chronic diabetes [11].

Mathieu et al. reported that vildagliptin monotherapy is associated with no weight gain and a low risk of hypoglycemia compared to a placebo. It lowered HbA1c like thiazolidinediones and acarbose when used alone, with sustained efficacy for up to two years [9]. Patients with T2DM who generally have a beta-cell reserve respond to vildagliptin. This is also indicated in subjects where the risk of hypoglycemia is high, such as the elderly, people with active careers, or those with concerns about weight gain. Hu et al. demonstrated that vildagliptin, given to healthy participants, did not alter insulin or glucose levels, suggesting it acts through the glucose-dependent pattern. It is a potent DPP4I that is tolerated at dosages ≤200 mg once daily (QD), displaying that the pharmacokinetics are roughly dose-proportional and show no signs of accumulation in the body even after repeated doses in healthy participants [12].

Hepatic and renal safety

Hepatic safety was comparable with both vildagliptin 50 mg twice daily (BD) doses [13]. Nor was there a higher incidence of pancreatitis, infections, or skin-related adverse events [14]. Vildagliptin 50 mg QD has proven safety and efficacy in severe renal impairment when hyperglycemia is uncontrolled with insulin [15] and in patients on dialysis [16]. It was effective in elderly patients (≥75 years) with moderate to severe renal impairment [17]. Vildagliptin 50 mg QD was effective when combined with insulin therapy in patients with severe renal impairment and long-term type 2 diabetes; reductions in HbA1c were similar to those previously reported among patients with recently developed type 2 diabetes with normal renal function. The incidence of hypoglycemia was similar to that of controls [15]. The DPP4 inhibition of vildagliptin was effective without any adverse effects in kidney transplant recipients with overt new-onset diabetes after transplantation (NODAT). It can therefore be considered an alternative therapeutic choice in this population [16].

Cardiovascular safety

The effect of vildagliptin (100 mg to 400 mg dose) in immediate-release (IR) forms and moxifloxacin 400 mg, the control drug, has been studied in healthy male volunteers. At the maximal daily dose of 100 mg or a four-fold higher dose (400 mg), vildagliptin had no apparent effect on cardiac repolarization (QT/QTc interval) or conduction (PR and QRS intervals) [18]. The upper bound for the placebo-adjusted mean changes from the two-sided 90% CI for the baseline in QT/QTc intervals in ECG evaluations met the protocol's predetermined limit of 58 ms and the current ICH E14 guidelines' limit of 510 ms [19]. In contrast, the placebo-adjusted mean QTcF intervals were below 5 ms in patients taking vildagliptin 100 mg at all periods except one hour after the dose. Five days of moxifloxacin 400 mg administration demonstrated a significant increase in the QTcF interval from baseline compared to placebo (p=0.007) but not a significant increase in the QTcB interval, according to maximum-change analysis. The QTcF and QTcB intervals did not significantly increase after a five-day course of treatment with vildagliptin 100 mg or 400 mg versus placebo [18].

Real-world evidence

**Figure 1:**
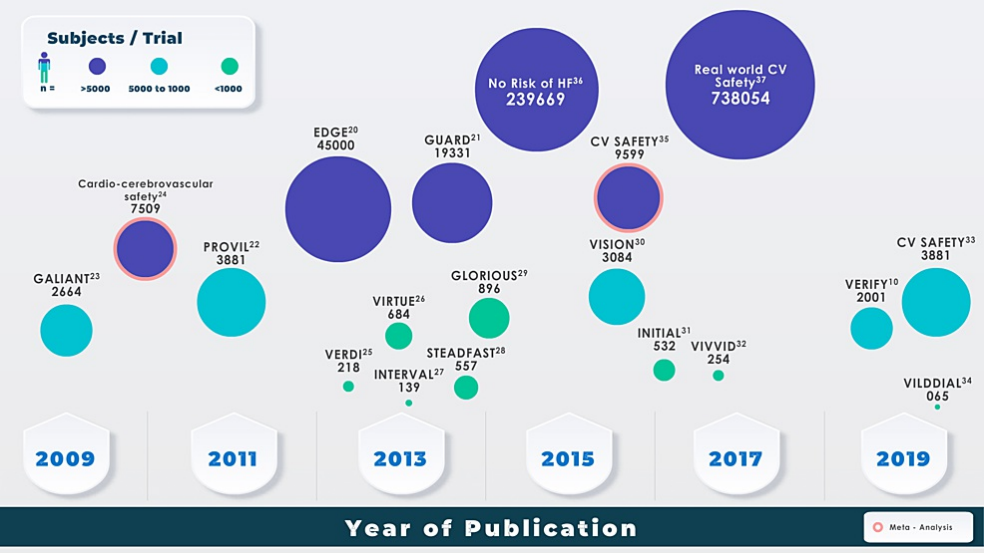
Landmark trials of vildagliptin over the years This Figure has been created by the authors to represent an overview of the vildagliptin trials [23-37], corresponding to their respective years of publication. The circles with pink circles are 'meta-analyses of vildagliptin'. HF: Heart failure, CV: Cardiovascular

Real-world studies such as EDGE, GUARD, and the German PROVIL show the efficacy and safety of vildagliptin in T2DM. The EDGE is a prospective, year-long, worldwide observational study that showed that vildagliptin was efficient and well-tolerated as a second-line agent. The HbA1c level reductions were greater in the Indian subjects who were a part of the study. Vildagliptin could therefore be considered a second-line agent in Indian patients not adequately controlled with monotherapy [20]. In the GUARD study, reductions in baseline HbA1c were observed irrespective of the patient’s age, BMI, or baseline HbA1c [21]. The German study PROVIL assessed the effect of combined vildagliptin and metformin and concluded that, compared to monotherapy with other oral agents, combined vildagliptin-metformin for six months resulted in a higher decrease in HbA1c levels [22]. Vildagliptin has been extensively studied over the years with a vast pool of patient data [23-37]. An overview of relevant studies of vildagliptin and the number of patients in each trial has been demonstrated in Figure 1.

Rationale for vildagliptin QD formulation

The nature of diabetes and its management are complex, requiring compliance-oriented care to simplify treatment. This may be done by making the drug regimen using QD combination pills less complicated [38]. The appeal of single pills as a QD formulation has increased as the burden of diabetes regimens increases, thus increasing adherence to the administration of antidiabetic agents.

Vildagliptin treatment given as a QD 100 mg SR formulation could fit the criteria to reduce pill burden. This SR formulation, given QD has the potential to provide glycemic control like the vildagliptin 50 mg BD formulation [39]. The bioequivalence of vildagliptin 100 mg SR QD has been established with vildagliptin 50 mg twice a day before meals (BID) at steady-state concentrations following oral administration. Joshi et al. demonstrated that vildagliptin QD SR 100 mg is bioequivalent to vildagliptin BD IR 50 mg. Vildagliptin SR 100 mg confers 80% DPP4 inhibition for 24 hours, enhancing glycemic control while reducing the frequency of drug intake [40].

The technology behind vildagliptin 100 mg SR tablets

**Figure 2:**
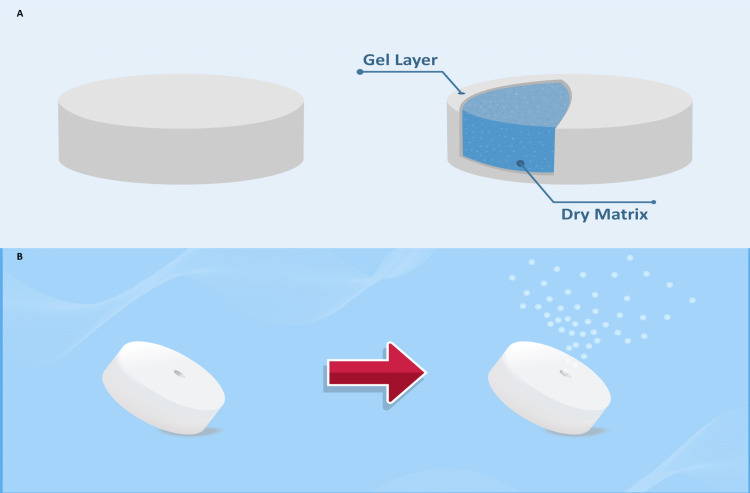
Osmotic drug delivery system A: Osmotic drug delivery formulation, B: Hydrophilic matrix release of the drug through a diffusion process This Figure has been created by the authors

The SR formulation of vildagliptin was developed to deliver 100 mg of vildagliptin for a longer time at a programmed rate by implementing a polymer matrix system that aids in the controlled release of the drug. Tablets with a hydrophilic matrix release the drug mostly through diffusion as opposed to an erosion process. The tablet's surface forms a gel when fluid comes in touch with it during the diffusion process (Figure 2A). The gel then establishes a diffusion barrier for the active pharmaceutical ingredient (API), which further enters a state of dissolution. Consequently, the gelation advances towards the tablet's core when the polymer matrix is hydrated (Figure 2B) [41].

The polymer matrix used in vildagliptin 100 mg SR QD is microcrystalline cellulose (MCC) PH 112. The MCC has been widely used in the formulation of multi-particulate and matrix tablet dosage forms for sustained-release drug delivery systems. Zero-order release profiles with MCC were reported. It has been demonstrated that this has no impact on the factors used to manufacture matrix tablets for drug release [42]. The polymer matrix system avoids peak-to-trough fluctuations and is not significantly affected by gastric pH and hydrodynamic conditions while having fewer side effects. Due to the regulated steady drug delivery, the normal peak and troughs of multiple dosing observed in the IR formulations can be completely bypassed [43-46].

Comparison of pharmacokinetic and pharmacodynamic parameters: vildagliptin 100 mg QD SR vs. vildagliptin 50 mg BID IR formulations

The pharmacodynamic profile of vildagliptin is assessed through its DPP-4 inhibition activity [47,48]. Similarly, the pharmacokinetic profile is assessed through plasma concentration-time, maximum concentration (Cmax), the area under the plasma concentration-time curve (AUC) from zero to 24 hour concentrations (AUC0-24), the time required to achieve maximal concentrations (Tmax), and T1/2 [48].

Joshi et al. observed that there were no apparent sequence, time, or treatment effects between QD vildagliptin SR 100 mg tablets and BD vildagliptin IR 50 mg tablets. Vildagliptin SR tablets showed substantially lower Cmax, and total drug exposures (AUC0-t and AUC0-inf) compared to vildagliptin IR tablets but higher Tmax and T1/2. These different max pharmacokinetic traits testify to the vildagliptin 100 mg QD sustained-release properties [40]. With sustained decreases in HbA1c levels and only a limited number of side effects, the pharmacokinetic and pharmacodynamic profiles of vildagliptin support a QD dosage [49].

Therapeutic efficacy of vildagliptin 100mg SR QD formulation

**Figure 3:**
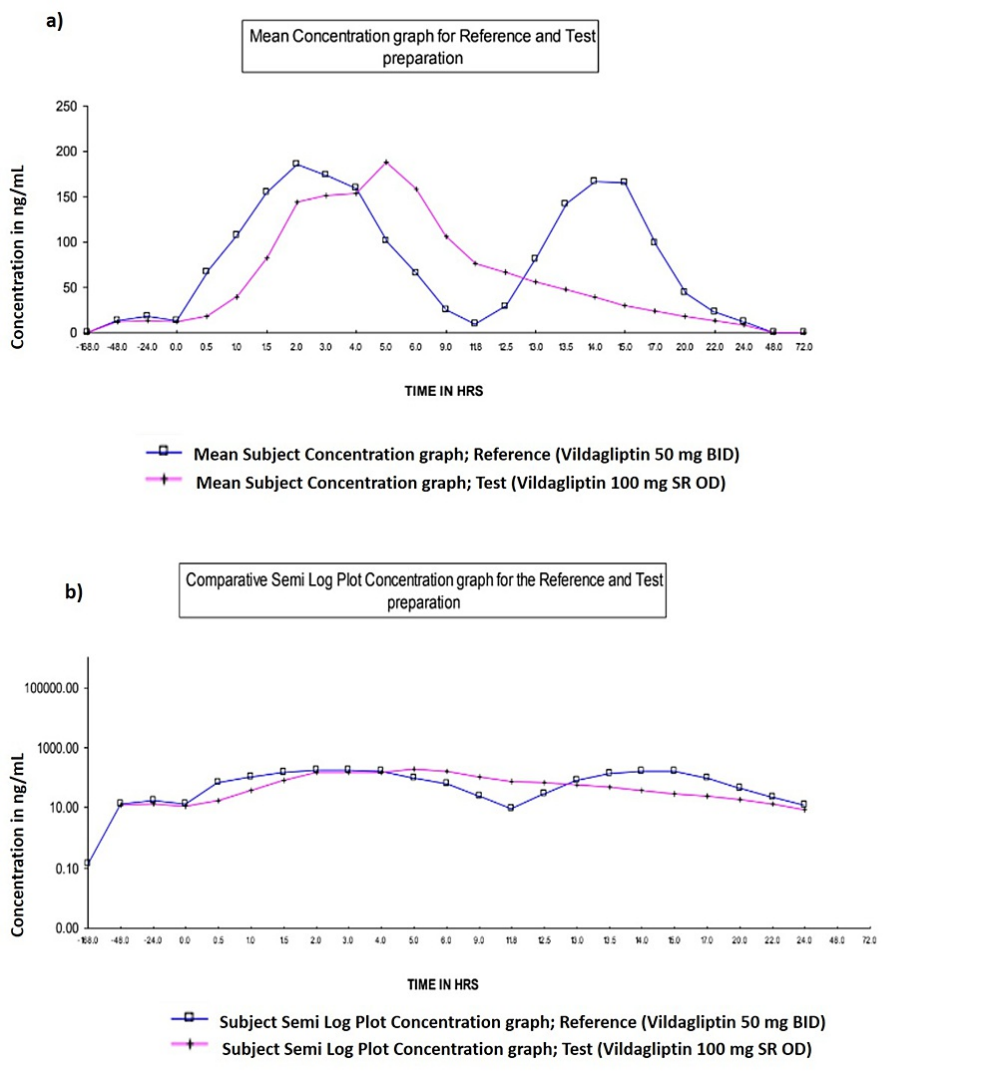
Comparison of mean and log plasma concentration versus time curve for vildagliptin SR 100 mg and 50 mg a) Mean plasma concentration vs. time curve for reference (R) and test (T) product; b) Log mean plasma concentration vs. time curve for reference (R) and test (T) product HRS: Hours Graphs used with permission from the article by Warrier et al. [40]

A randomized and open-labeled study assessing the efficacy and safety of vildagliptin 100 mg SR concluded that the DPP-4 inhibition percentage (0 to 24 hours) was 90% with vildagliptin SR 100 mg and 91.05% with vildagliptin 50 mg [40]. When combined with metformin 1000 mg, vildagliptin 100 mg SR QD dose was as efficient and safe as a 50 mg BD dose in terms of lowering HbA1c, fasting plasma glucose, and postprandial glucose [41]. According to the bioequivalence criterion of CDSCO, India, the three brands of vildagliptin 50 mg (Galvus®, Jalra®, and Zomelis®) were bioequivalent in a single-dose trial in healthy Indian volunteers in fasting conditions [49]. A randomized, two-sequence, multiple-dose, crossover, and open-labeled study in normal adult male subjects evaluated that oral vildagliptin SR 100 mg was bioequivalent to vildagliptin 50 mg BD in terms of rate and extent of absorption under fasting conditions. It was observed that the geometric mean percentage of DPP-4 inhibition (0 to 24 hours) was 90% with vildagliptin SR 100 mg and 91.05% with vildagliptin 50 mg. In addition, the weighted average percentage of DPP-4 inhibition (0 to 24 hours) was 86.15% with vildagliptin SR 100 mg and 85.35% with vildagliptin 50 mg. The mean plasma concentration of vildagliptin 50 mg BD was at its peak of 200 ng/mL at 2.0 hours, dropped drastically at 11.8 hours and then increased again to 150 ng/mL at 15.0 hours, sustained to 17.0 hours. In contrast, the mean plasma concentration for vildagliptin SR 100 mg increased steadily from 150 ng/mL at 2.0 hours and then reached a peak of 200 ng/mL at 5.0 hours, after which a steady decrease with no dramatic drop was observed [40]. The mean and log plasma concentration versus time curve for vildagliptin SR 100 mg and 50 mg products are given in Figure 3.

Safety reporting of vildagliptin 100mg SR QD

The periodic safety update report for marketed medications (PSUR) was created as a standalone document to enable a cyclical, thorough evaluation of the global safety data of a marketed drug or biological product. A crucial pharmacovigilance tool may be PSUR. 

The PSUR data of vildagliptin 100 mg SR QD, can be calculated with the following formula:

Patient-time exposure in patient treatment year (PTY) = total volume of unit sold*strength/(daily defined dose(DDD) x 365)

Considering the launch of vildagliptin 100 mg SR QD in April 2021 and applying the data in the formula, the values come to over 3000 patient years.

Patient-time exposure in patient treatment year (PTY) = 1109322*100/(100*365)=3039.238

## Conclusions

Vildagliptin is an effective and well-tolerated molecule throughout the diabetes continuum. Studies performed in India show its efficacy and safety in the presence of other comorbidities and diabetic complications, and across a spectrum of age groups. Both vildagliptin SR 100 mg QD and vildagliptin 50 mg BD showed similar DPP4 inhibition for 24 hours. The bioequivalence and similar pharmacokinetic/pharmacodynamic profiles of vildagliptin 100 mg QD SR with the BID dosage pharmacodynamic profile correlate with its pharmacokinetic profile. Its distinct characteristics make the vildagliptin 100 mg QD SR formulation a safe and well-tolerated medication that can improve patient compliance compared to the vildagliptin 50 mg BD IR formulation. The single dose of 50 mg vildagliptin provides DPP4 inhibition of ≥80% for 12 hours. However, a single bolus of 100 mg vildagliptin confers the same DPP4 inhibition for 15 to 16 hours. Reduced dosing frequency, fewer side effects, steady and prolonged release of medications, and enhanced patient compliance are some of the reported benefits of the vildagliptin QD SR formulation.
